# Supplementary material for: Composition and Structure of Arabidopsis thaliana Extrachromosomal Circular DNAs Revealed by Nanopore Sequencing
Source: Plants (Basel). 2023 May 30;12(11):2178. doi: 10.3390/plants12112178 (PMC10255303; doi:10.3390/plants12112178)
Supplement: Supplementary file 1 [file plants-12-02178-s001.zip › plants-2404263-supplementary.pdf]

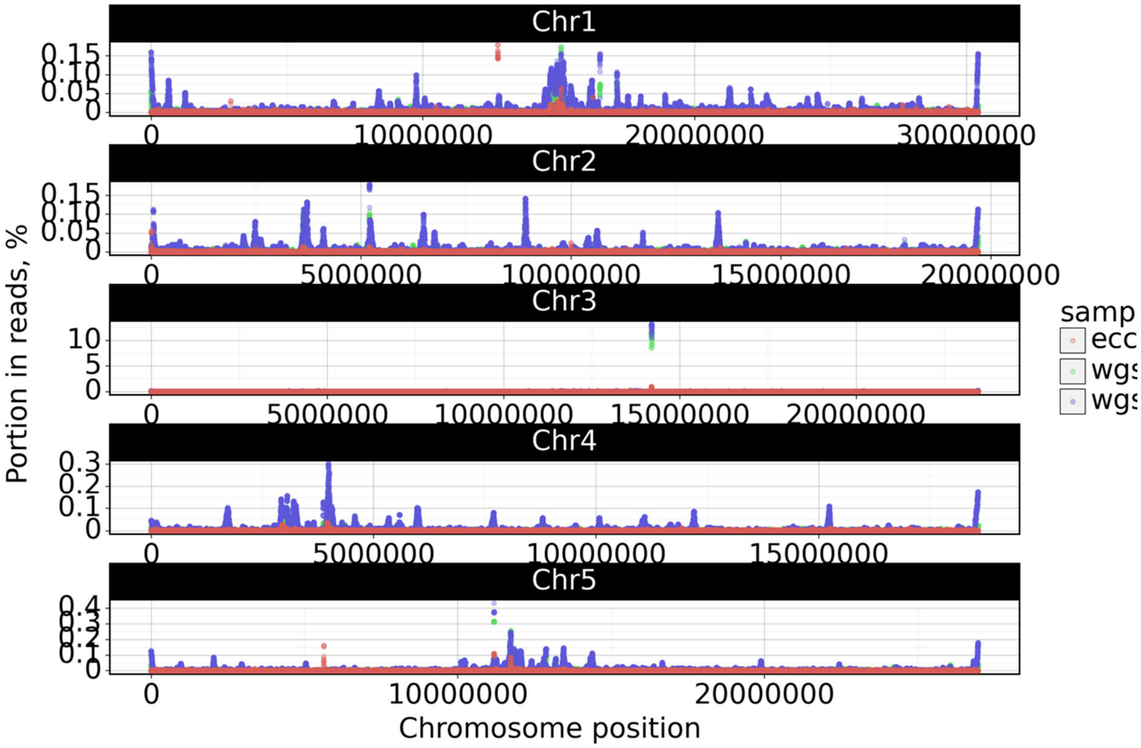

Figure S1. Mapping of selected concatemer reads with 2 or more monomers obtained from Nanopore sequencing eccDNA-enriched ddm1 DNA (red) as well as whole-genome sequencing of Col-0 (green) and ddm1 (blue) plants.

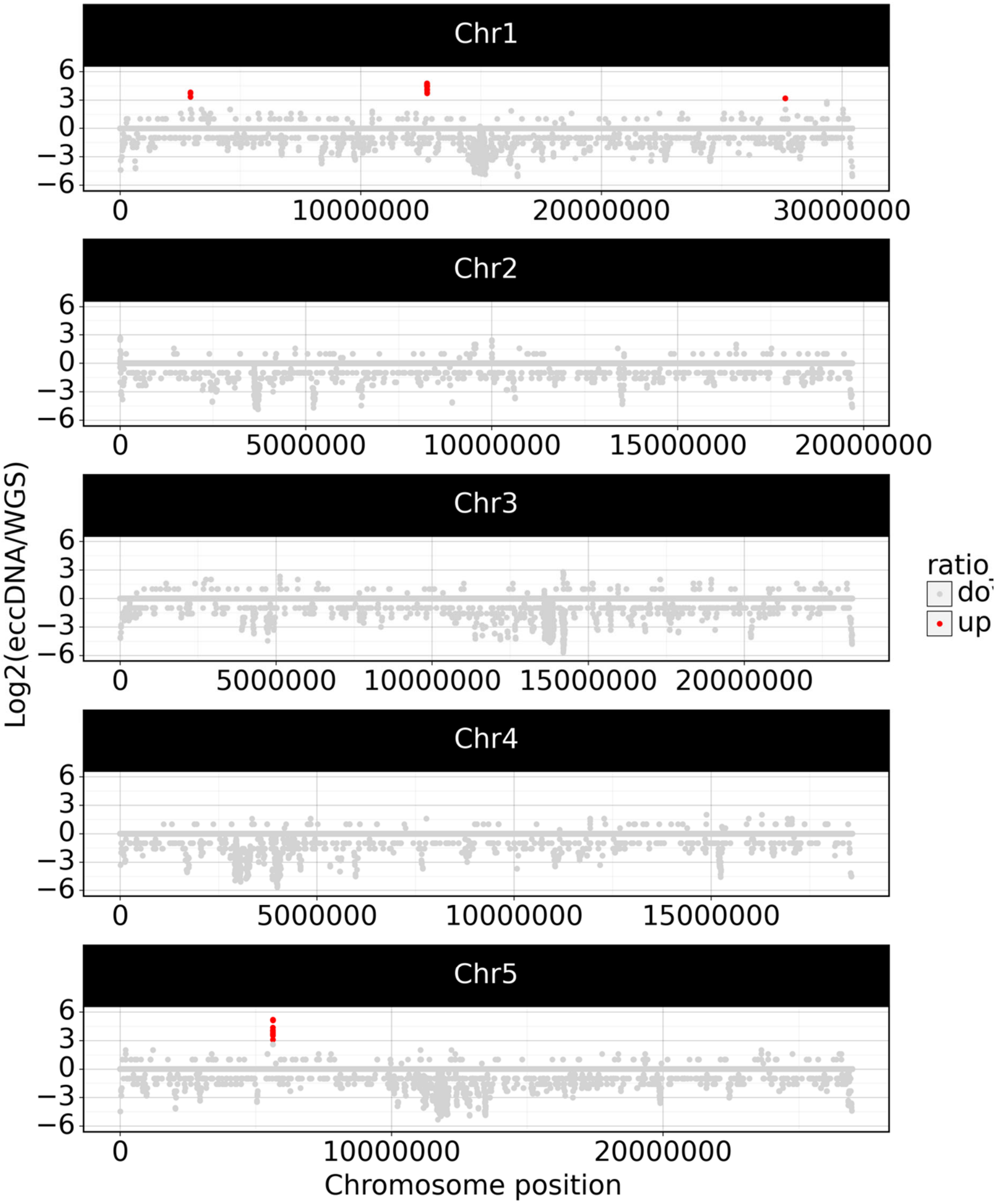

Figure S2. EccDNA read coverage of genome loci. Dots indicate log2 ratio between number of concatemer reads of eccDNA and WGS samples for *ddm1* plants. Red and grey colors show regions with the log2 ratio >3 or =< 3, respectively.

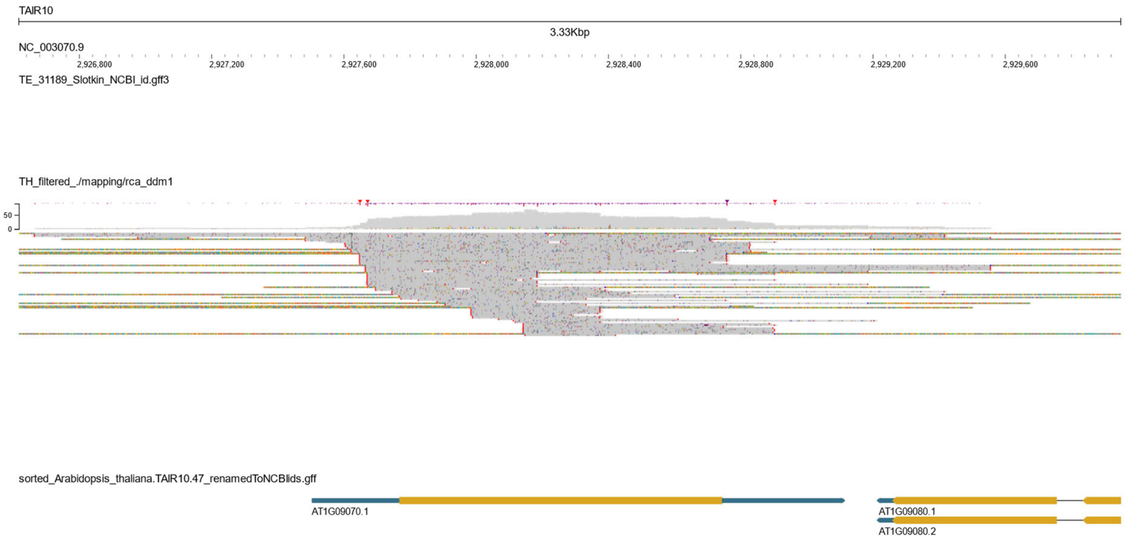

Supplementary Figure S3. Coverage of AT1G09070 gene (Chr1:2,927,502..2,929,107) by Nanopore concatemer reads from eccDNA-enriched DNA of ddm1.

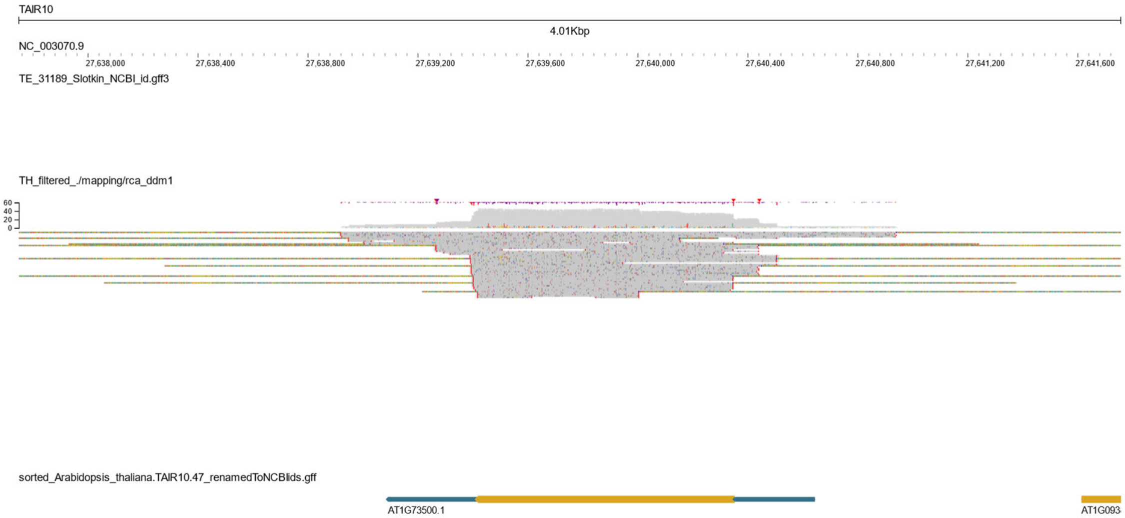

Figure S4. Coverage of AT1G73500 gene (Chr1: 27,637,748..27,641,755) by Nanopore concatemer reads from eccDNA-enriched DNA of ddm1.

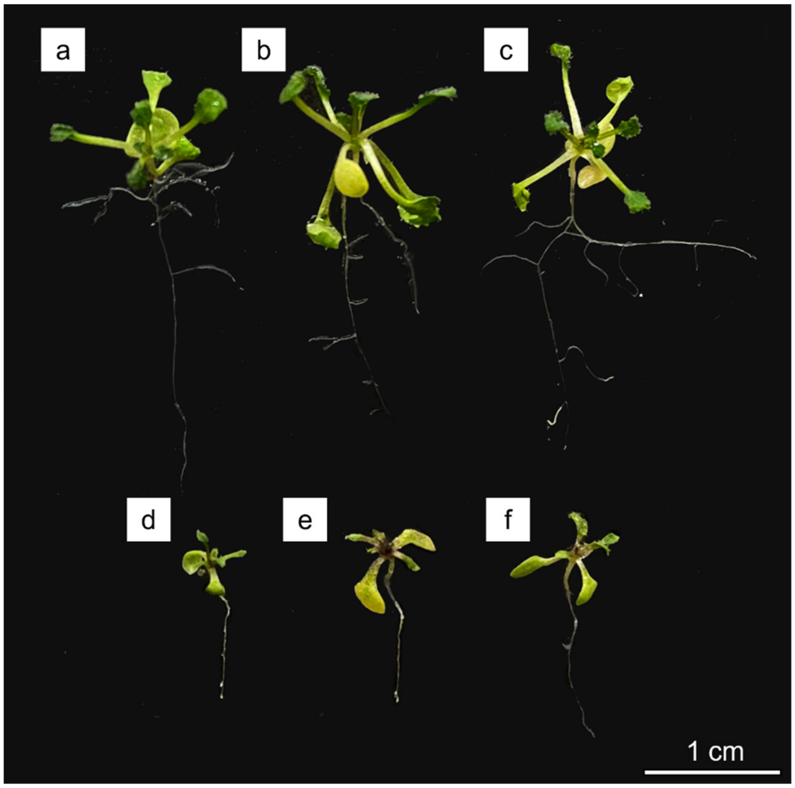

Figure S5. Inhibition of *A.thaliana* Col-0 growth and development under toxin treatment (zebularine and α-amanitin**).** a, b, c – control plants grown *in vitro*; d, e, f – plants grown on ½ MS supplemented with zebularine and α-amanitin.

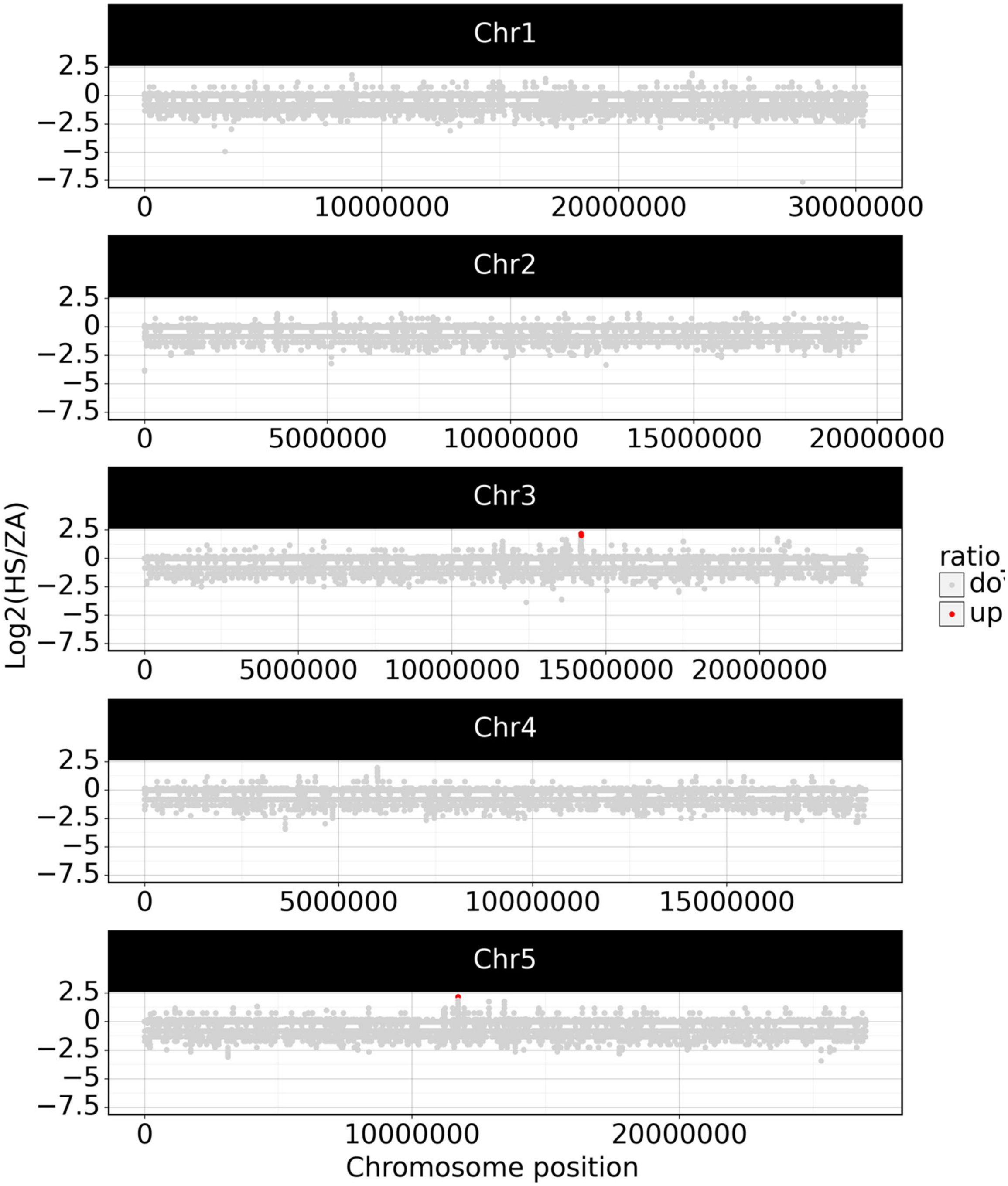

Figure S6. EccDNA read coverage of genomic loci in ZA vs K sample. Dots indicate log2 ratio between number of concatemer reads of eccDNA for ZA vs K sample. Red and grey colors show regions with the log2 ratio >=2 or < 2, respectively.

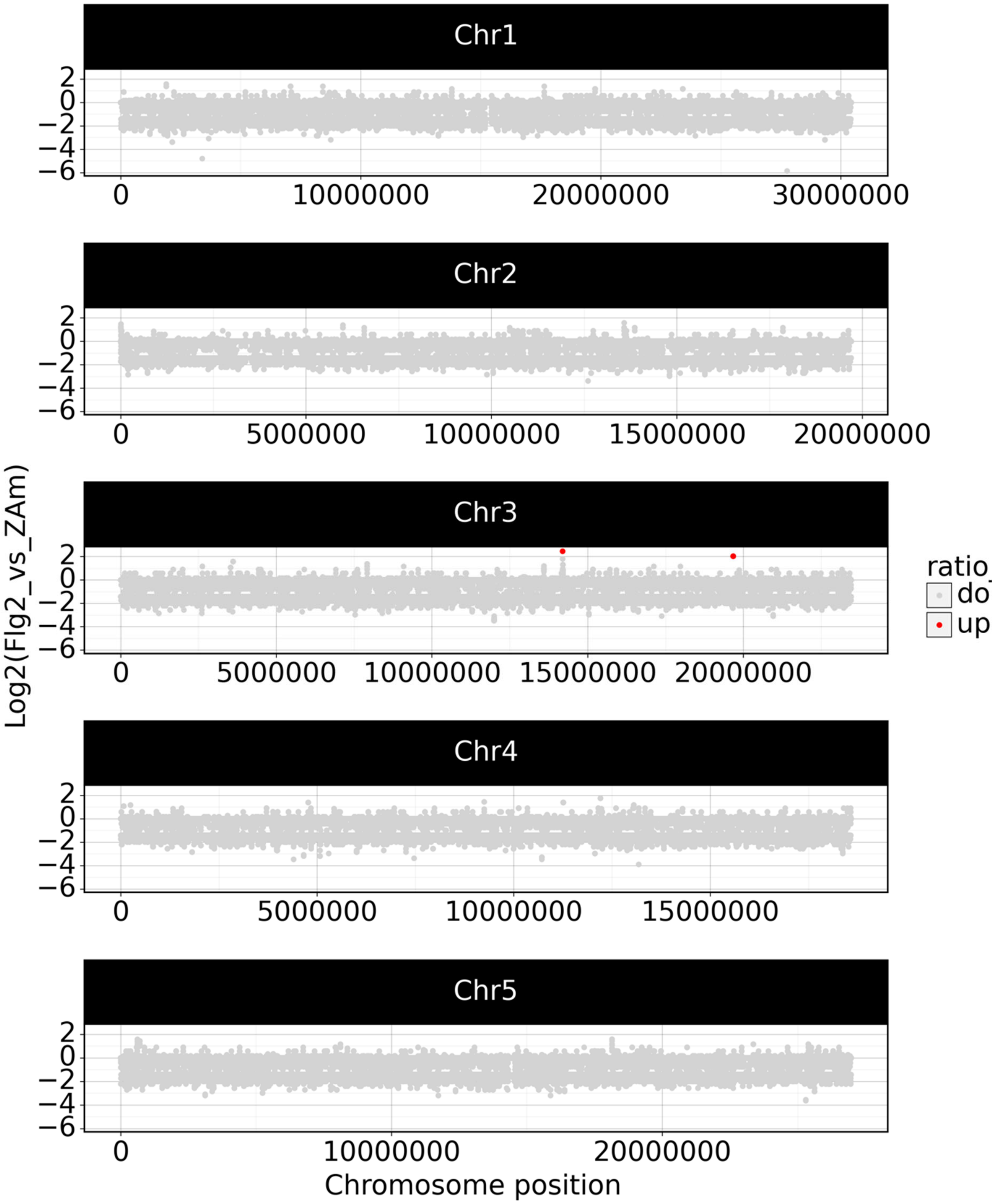

Figure S7. EccDNA read coverage of genomic loci from Flg samples (replicate 1). Dots indicate log2 ratio between number of concatemer reads of eccDNA for Flg vs ZA sample. Red and grey colors show regions with the log2 ratio >=2 or < 2, respectively.

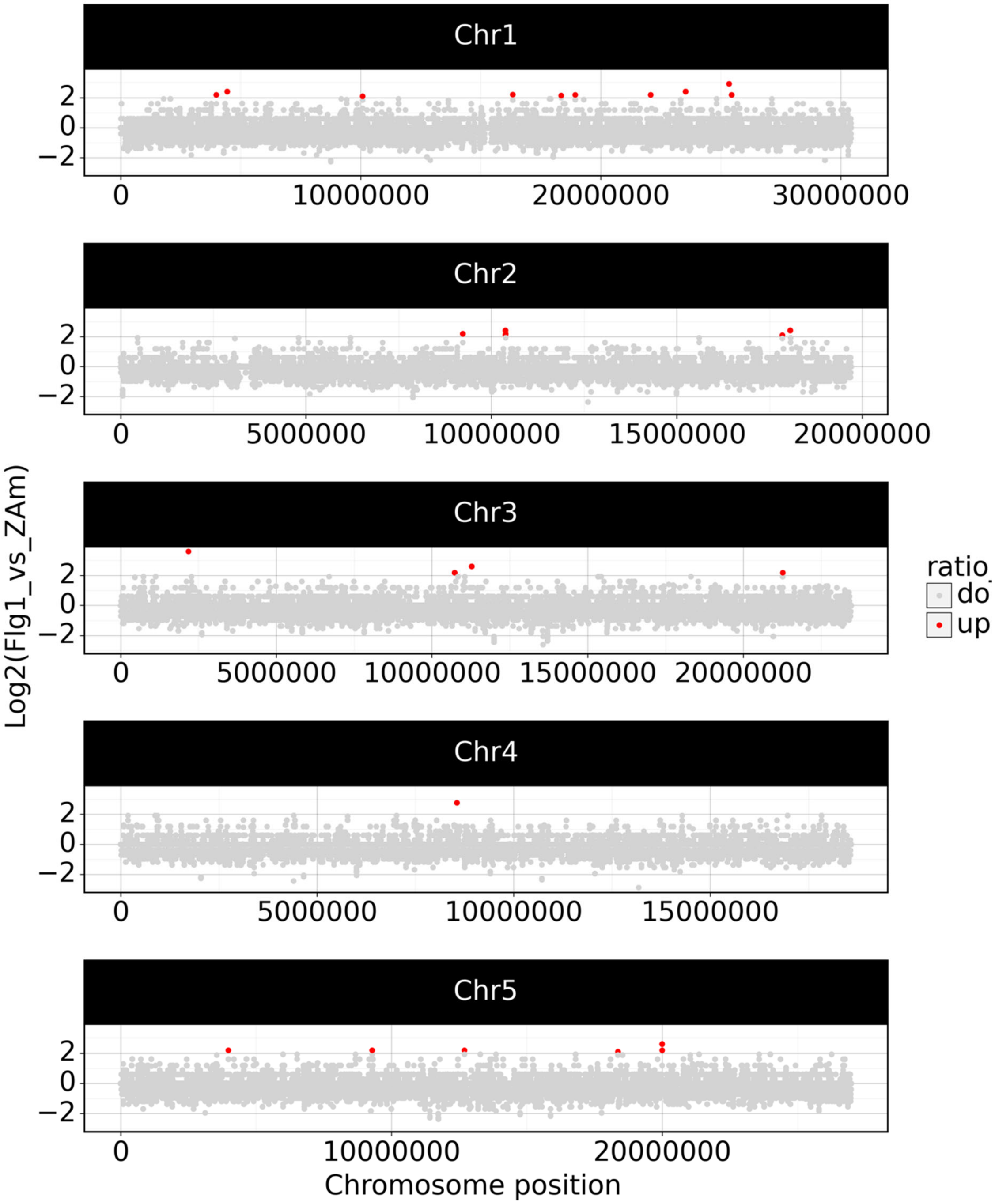

Figure S8. EccDNA read coverage of genomic loci from Flg samples (replicate 2). Dots indicate log2 ratio between number of concatemer reads of eccDNA for Flg vs ZA sample. Red and grey colors show regions with the log2 ratio >=2 or < 2, respectively.

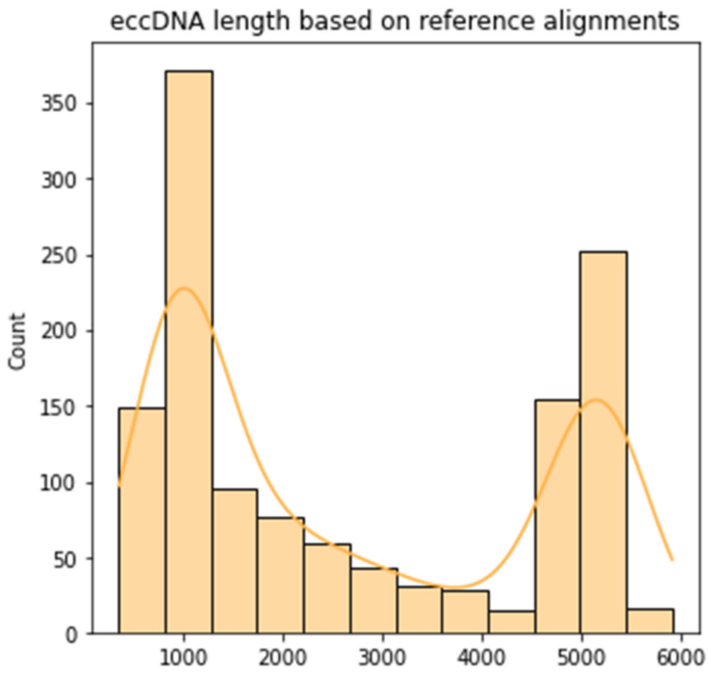

Figure S9. The histogram showing number of EVD eccDNAs of different length.
